# Inhibiting glycosphingolipids alleviates cardiac hypertrophy by reducing reactive oxygen species and restoring autophagic homeostasis 

**DOI:** 10.3389/fphar.2024.1409625

**Published:** 2024-10-01

**Authors:** Chunxin Jiang, Menglei Tan, Lunmeng Lai, Yanping Wang, Zijun Chen, Qing Xie, Yunsen Li

**Affiliations:** ^1^ Jiangsu Key Laboratory of Infection and Immunity, Institutes of Biology and Medical Sciences, Suzhou Medical College of Soochow University, Soochow University, Suzhou, China; ^2^ College of traditional Chinese medicine, Shanghai University of Traditional Chinese Medicine, Shanghai, China

**Keywords:** cardiac hypertrophy, glycosphingolipid, lactosylceramide, reactive oxygen species, mitochondrial autophagy

## Abstract

**Introduction:**

Cardiac hypertrophy is a compensatory stress response produced by a variety of factors, and pathologic hypertrophy can lead to irreversible, severe cardiac disease. Glycosphingolipids (GSLs) are vital constituents of cells, and changes in their content and composition are important factors causing mitochondrial dysfunction in diabetic cardiomyopathy; however, the relationship between GSLs expression and cardiac hypertrophy and specific mechanisms associated with it are not clear.

**Methods:**

Here, using male C57BL/6 mice, we performed aortic arch reduction surgery to establish an animal model of pressure overload cardiac hypertrophy. In addition, phenylephrine was used *in vitro* to induce H9c2 cells and neonatal rat left ventricular myocytes (NRVMs) to establish a cellular hypertrophy model.

**Results:**

Mass spectrometry revealed that the composition of GSLs was altered in pressure overload-induced hypertrophied mouse hearts and in stimulated hypertrophied cardiomyocyte cell lines. Specifically, in both cases, the proportion of endogenous lactosylceramide (LacCer) was significantly higher than in controls. Inhibition of GSL synthesis with Genz-123346 in NRVMs reduced cell hypertrophy, as well as fibrosis and apoptosis. By Western blotting, we detected decreased intracellular expression of Sirt3 and elevated phosphorylation of JNK after phenylephrine stimulation, but this was reversed in cells pretreated with Genz-123346. Additionally, increased protein expression of FoxO3a and Parkin, along with a decreased LC3-II/I protein ratio in phenylephrine-stimulated cells (compared with unstimulated cells), indicated that the mitochondrial autophagy process was disrupted; again, pretreatment with Genz-123346 reversed that.

**Discussion:**

Our results revealed that changes in GSLs in cardiomyocytes, especially an increase of LacCer, may be a factor causing cellular hypertrophy, which can be alleviated by inhibition of GSLs synthesis. A possible mechanism is that GSLs inhibition increases the expression of Sirt3 protein, scavenges intracellular reactive oxygen species, and restores mitochondrial autophagy homeostasis, thereby lessening cardiomyocyte hypertrophy. In all, these results provide a new perspective for developing drugs for cardiac hypertrophy.

## 1 Introduction

Cardiac hypertrophy is initially an adaptive, compensatory response to hemodynamic stress (Berenji et al., 2005; Frey and Olson, 2003); however, chronic stimulation of the heart (e.g., myocardial injury or valvular disease) can lead to pathologic hypertrophy, which impairs contractility and ultimately develops into heart failure. Pathological hypertrophy of the heart can be caused by a variety of factors, such as neurohormonal overactivation, increased oxidative stress, inflammatory signals, and mechanical stress. These stimuli directly or indirectly increase the production of reactive oxygen species (ROS) and the accumulation of metabolic intermediates, which trigger intracellular signaling pathways, such as the mitogen-activated protein kinase (MAPK), Ca^2+^-related, and Wnt signaling pathways; ultimately, these pathways lead to mitochondrial dysfunction, cell fibrosis, and death (Nakamura and Sadoshima, 2018).

Oxidative stress results from dysregulation of the balance between ROS production and the body’s intrinsic antioxidant system (Nordberg and Arnér, 2001). Excessive accumulation of ROS disrupts cellular homeostasis and causes mitochondrial dysfunction (Chakrabarti et al., 2014; Fandy et al., 2014), which plays a deleterious role in a variety of cardiac diseases, including cardiac hypertrophy. Inhibition of nuclear protein BRD4 expression can alleviate pathological cardiac hypertrophy by scavenging ROS to suppress fibrosis and inflammation (Zhu et al., 2020). Autophagy, a highly conserved process widely found in eukaryotic cells, degrades misfolded and senescent proteins and removes damaged organelles through lysosome-mediated pathways (Bernardo et al., 2010; Wang et al., 2012). Furthermore, autophagy is an important mechanism for maintaining myocardial structure and cellular homeostasis; however, excessive activation of autophagy also damages organelles and releases apoptosis-related factors.

Glycosphingolipids (GSLs), a lipid component distributed in cell membranes, is one of the most abundant glycolipids in the human body. It acts as a second messenger to regulate a wide range of phenotypes (Hannun and Obeid, 2018) and is involved in not only basic cellular processes such as growth, development, and differentiation but also pathological processes such as tumors and cardiovascular diseases. GSLs-centered signaling pathways play a role in the regulation of such processes as inflammation, oxidative stress, apoptosis, autophagy, and mitochondrial dysfunction (Chatterjee et al., 2021). Furthermore, a variety of diseases have been shown to be associated with dysregulated metabolism of GSLs. For example, accumulation of lactosylceramide (LacCer) in the heart of type I diabetic mice is a major contributor to mitochondrial dysfunction (Novgorodov et al., 2016). Serum levels of C16:0-LacCer are significantly higher in children with inflammatory bowel disease than in healthy children, and the increase is most pronounced in the sera of children with Crohn disease; thus, LacCer has been suggested as a biomarker for inflammatory bowel disease (Filimoniuk et al., 2020). Targeting GSLs synthase may be an important tool in the treatment of these diseases.

The pathogenesis of cardiac hypertrophy and related signaling pathways is intricate, and currently available therapies still fail to achieve satisfactory results; thus, it is crucial to identify new signaling targets and explore promising therapeutic approaches. Adding exogenous LacCer to cardiomyocytes promotes cellular hypertrophy through increased ROS production and activation of the ERK1/2 signaling pathway (Mishra and Chatterjee, 2014) and is specific compared with other GSLs. However, the specific relationship between GSLs and pressure overload cardiac hypertrophy is unclear.

In this study, we analyzed the changes of endogenous GSLs with different glycan moieties in the hearts of mice with pressure overload-induced cardiac hypertrophy. Our preliminary study on the effects of GSLs inhibition in cardiac hypertrophy provided insight into the mechanism of action of GSLs in this model. Specifically, GSLs inhibition increases the expression of Sirt3 protein, scavenges intracellular reactive oxygen species, and restores mitochondrial autophagy homeostasis, thereby lessening cardiomyocyte hypertrophy.

## 2 Materials and methods

### 2.1 Reagents

Phenylephrine (P1240000), DEAE Sephadex A-25 (A25120), and Triton X-100 (T9284) were purchased from Sigma-Aldrich (St. Louis, MO, USA). Type II collagenase (05401151001) was purchased from Roche (Basel, Switzerland). GSL synthesis inhibitor Genz-123346 (HY12744) was purchased from MCE (Shanghai, China). DMEM-F12 (11320033) was purchased from Gibco (Grand Island, NY, United States). High-sugar DMEM (L110KJ) and trypsin (S310JV) were purchased from BasalMedia (Shanghai, China). Fetal bovine serum (FBS) (C04001) was purchased from VivaCell (Shanghai, China). Penicillin-streptomycin solution (BL505A) was purchased from Biosharp (Guangzhou, China). 5-Brdu (ED1100) was purchased from Boster (Wuhan, China). RNA Rapid Extraction Kit (U10018) was purchased from Coyobo (Suzhou, China). HiScript Ⅲ RT SuperMix for quantitative PCR (qPCR) kit (R323-01), SYBR qPCR Master Mix (Q712-02), and 180 kDa Prestained Protein Marker (MP102-01) were purchased from Vazyme (Nanjing, China). 10–250 kDa Prestained Protein Marker (WJ103) was purchased from Yamei (Shanghai, China). SDS-PAGE gel preparation kit (P0012A), RIPA (P0013B), 100 mM PMSF (st506), JC-1 mitochondrial membrane potential kits (C2003S), ROS detection kit (S003s), BCA protein concentration measurement kit (P0012), and DAPI (C1002) were purchased from Beyotime (Shanghai, China). 5 × SDS-PAGE protein loading buffer (20315ES20) was purchased from Yeasen (Shanghai, China). Non-fat powdered milk (A600669-0250) was purchased from BBI (Crumlin, United Kingdom). PVDF membranes (0.45 μm, IPVH00010; 0.22 μm, ISEQ00010) were purchased from Millipore (Boston, MA, United States). Paraformaldehyde fixative (G1101) was purchased from Servicebio (Wuhan, China). High-sig ECL Western blotting substrate (180–501) was purchased from Tanon (Shanghai, China). Cell Counting Kit-8 (CCK8) (FD3788), Femto ECL Kit (FD8030), and protein phosphatase inhibitor mixture (FD1002) were purchased from FuDe (Hangzhou, China). Primary antibodies for anti-Sirt3 (5490 s), anti-JNK (9252 s), anti-phospho-JNK (4668 s), anti-FoxO3a (2497 s), anti-caspase3 (9662 s), and anti-cleaved caspase3 (9664 s) and second antibody for HRP anti-R IgG (7074 s) were purchased from Cell Signaling Technology (Danvers, MA, United States). Primary antibodies for anti-collagen I (ab270993), anti-collagen III (ab184993), and anti-α-smooth muscle actin (anti-α-SMA; ab184675) were purchased from Abcam (Cambridge, United Kingdom). Primary antibodies for anti-NPPA (27426-1-AP) and anti-MYH7 (22280-1-AP) were purchased from Proteintech (Chicago, IL, United States). Anti-LC3B antibody (L7543) was purchased from Sigma-Aldrich, anti-ANP antibody (sc-515701) was purchased from Santa Cruz (Dallas, TX, USA), and HRP anti-M IgG (Rs0001) was purchased from Immunoway (Newark, NJ, United States). Primary antibodies for anti-GAPDH (380626) and anti-Parkin (381626) were purchased from ZENBIO (Chengdu, China). DyLight 488-labeled goat-anti-mouse IgG (H + L) (5230–0391) was purchased from Seracare (Milford, MA, United States).

### 2.2 Animals

The C57BL/6 male mice (3–4 months old) used for our animal model were purchased from Guangdong Medical Laboratory Animal Center. The environment of the facility was maintained at a temperature of 22–26°C, humidity of 40%–60%, and 12-h light/dark cycle. Mice were allowed to enter the experimental facility for 1 week to acclimatize before the start of the experiment. The 1–3 days Sprague-Dawley (SD) neonatal rats used to extract primary cardiomyocytes were purchased from Jihui Laboratory Animal Breeding Co (Shanghai, China). The animal experiments followed the guidelines of the Implementing Rules for the Management of Medical Animal Experiments and were approved by the Animal Policy and Welfare Committee of Soochow University.

### 2.3 Transverse aortic constriction (TAC) surgery

Transverse aortic constriction (TAC) surgery was implemented as relevant literature (Kandalam et al., 2011; Zhabyeyev et al., 2013; Wang et al., 2018). Mice with normal cardiac function were anesthetized with a mixture of isoflurane gas. The thoracic cavity was opened, and the aortic arch was ligated with a ligation line that was tied tightly. The thoracic cavity was then sutured layer by layer after the mice were stabilized. In sham group mice, the ligation was not performed.

### 2.4 Extraction of neutral GSLs

The extraction of neutral GSLs was performed as described in the literature (Li et al., 2008). Briefly, homogenized mouse heart tissue or about 2.4 × 10^7^ cells were taken in a glass test tube and extracted four times with solution A (methanol: chloroform = 1:1, v/v) by sonication, followed with solution B (isopropanol: n-hexane: water = 55:25:20, v/v/v) by sonication. The supernatants were pooled and dried. The dried total GSLs were separated in a DEAE Sephadex A-25 separation column, and the neutral GSLs were eluted with solution C (chloroform: methanol: water = 30:60:8, v/v/v) and dried. Next, the GSLs were derivatized with iodomethane in alkaline conditions (Zhu et al., 2014). Premethylated GSLs were injected into an LTQ-XL mass spectrometer (Thermo Fischer Scientific, Waltham, MA, United States) for detection.

### 2.5 H9c2 cell culture and treatments

H9c2 cells were purchased from Punosei Life Sciences (Wuhan, China) and cultured in high-sucrose DMEM containing 10% FBS and 1% penicillin-streptomycin at 37°C in a humidified incubator with 5% CO_2_. Control and phenylephrine-treated groups were also set up. After H9c2 cells were plated overnight, they were switched to a low-serum medium containing 2% FBS for culture, while the phenylephrine group was stimulated with 20 μM or 100 μM phenylephrine for 48 h.

### 2.6 Extraction and culture of neonatal rat left ventricular myocytes (NRVMs)

SD neonatal rats (1–3 days old) were euthanized by CO_2_ inhalation. Hearts were cut into 1-mm^3^ pieces and washed twice with D-Hank’s buffer, then centrifuged at 900 rpm for 5 min. The supernatant was discarded, and the tissue was digested with 2 mL digestive enzyme working solution (5 μL type II collagenase master mix per heart, diluted with D-Hank’s buffer) for 10 min at 37°C in a water bath, with shaking every 3 min. The digestion was repeated 5–6 times until the tissue mass disappeared. Except for the first time, the remaining supernatants were collected in DMEM-F12 containing 15% FBS to terminate the digestion, and all cell suspensions were collected and centrifuged at 900 rpm for 5 min. That supernatant was discarded, and the cells were resuspended with DMEM-F12 and filtered through a 70-μm cell sieve. Then the cells were spread in cell culture dishes, which were placed in a humidified incubator at 37°C with 5% CO_2_ for 2 h to remove adherent fibroblasts. Cardiomyocytes were collected in centrifuge tubes and centrifuged, then resuspended in DMEM-F12. Fibroblast growth was inhibited by adding 5-Brdu at a final concentration of 100 μM. Finally, the cardiomyocytes were spread in well plates at the appropriate density. NRVMs were cultured with DMEM-F12 containing 10% FBS and 1% penicillin-streptomycin at 37°C in a humidified incubator with 5% CO_2_. They were divided into control, phenylephrine, and phenylephrine + Genz-123346 groups. The cells in the GSL inhibition group were pretreated with Genz-123346, and after 60 h, all except the control group were stimulated by adding 100 μM phenylephrine for 48 h.

### 2.7 CCK-8 assay

NRVMs were cultured in 96-well plates with different concentrations of Genz-123346 and incubated for 60 h. Then 10 μL of CCK-8 reagent was added to each well according to the manufacturer’s instructions, and the incubation was continued for 4 h. The absorbance values were measured at 450 nm using a multifunctional enzyme labeling instrument (Tecan, Infinite M200pro, Switzerland).

### 2.8 RNA extraction and real-time qPCR analysis

Total RNA was extracted using the RNA Rapid Extraction Kit according to the manufacturer’s instructions. The RNA (1,000 ng) was reverse transcribed into cDNA using HiScript Ⅲ RT SuperMix for qPCR kit. Real-time fluorescent qPCR was performed using SYBR Green PCR Mix with the ABI 7500 Sequence Detection System (Thermo Fisher Scientific, Carlsbad, CA, United States); the reaction program was 95°C for 30 s, 40 cycles at 95°C for 5 s, and 60°C for 30 s. The abundance of each target gene mRNA was normalized to the GAPDH gene and calculated using the 2^−ΔΔ^CT method. The sequences of the rat primers used in the experiments are listed in Table 1.

**TABLE 1:** Sequences of primers used for RT-PCR.

Gene	Forward primers	Reverse primers
GAPDH	5′-AGG​TCG​GTG​TGA​ACG​GAT​TT-3′	5′-TGT​AGA​CCA​TGT​AGT​TGA​GGT​CA-3′
ANP	5′-AGC​CGA​GAC​AGC​AAA​CAT​CA-3′	5′-TAC​CGG​CAT​CTT​CTC​CTC​CA-3′
BNP	5′-AGC​TCT​CAA​AGG​ACC​AAG​GC-3′	5′-TCC​GGT​CTA​TCT​TCT​GCC​CA-3′
β-ΜΗC	5′-CCT​CGC​AAT​ATC​AAG​GGA​AA-3′	5′-TAC​AGG​TGC​ATC​AGC​TCC​AG-3′

### 2.9 Western blot analysis

After treatment with phenylephrine or Genz-123346, cells were collected and lysed with pre-cooled RIPA lysate containing protease and protein phosphatase inhibitors, and centrifuged at 12,000 rpm for 15 min at 4°C. The total protein content of the supernatant was quantified using a BCA protein assay kit, and then the protein samples were boiled with 5 × SDS loading buffer for 5 min. Protein samples were separated by SDS-PAGE and transferred to a PVDF membrane, which was blocked with 5% non-fat milk powder (prepared with 1× TBST) for 1 h at room temperature. The blocking solution was discarded, and the membrane was washed 3 times with 1 × TBST for 5 min each time. We incubated the primary antibody overnight at 4°C, and the membrane was washed 3 times with 1 × TBST for 10 min each time. The membrane was incubated with HRP-labeled secondary antibody for 1–2 h at room temperature and washed 3 times with 1 × TBST for 10 min each time. Protein expression was detected using an ECL luminescent solution and a chemical spectrophotometric image analysis system (Tanon, Shanghai, China). Protein expression was quantified using the ImageJ program.

### 2.10 Immunofluorescence staining

After treatment, cells were washed 3 times with PBS, fixed with 4% paraformaldehyde for 20 min, permeabilized with 0.5% Triton-100 for 20 min, and blocked with 2% BSA for 30 min at room temperature. Cells were then incubated with α-SMA primary antibody overnight at 4°C, washed 3 times with PBS, and incubated with DyLight 488-labeled goat-anti-mouse IgG at room temperature for 1 h. Then they were incubated with DAPI staining solution at room temperature for 8 min. Stained cells were analyzed with a fluorescence microscope (Nikon, Japan, CKS53) at ×200 magnification.

### 2.11 JC-1 staining

The mitochondrial membrane potential of the cells was detected using the Enhanced Mitochondrial Membrane Potential Assay Kit. Cells were stained with freshly prepared JC-1 staining working solution after completion of the treatment with phenylephrine or Genz-123346. The cells were incubated at 37°C for 20 min and then washed with JC-1 staining buffer to remove excess dye. Stained cells were analyzed with a laser confocal microscope (Nikon, Japan, A1) at ×200 magnification.

### 2.12 ROS assay

Intracellular ROS levels were detected using the DCFH-DA fluorescent probe. Cells were treated with phenylephrine or Genz-123346, incubated with DCFH-DA staining solution at 37°C for 20 min in the dark, and then washed three times with DMEM-F12. The fluorescence value of DCF was detected by an enzyme marker at 488 nm excitation wavelength and 525 nm emission wavelength. The DCF fluorescence intensity was observed with a laser confocal microscope at ×200 magnification.

### 2.13 Statistical analysis

All experiments were repeated at least three times. Statistical analysis was performed using GraphPad Prism 9.1.1. Unpaired *t*-test was used to compare the mean values between the two samples, and the one-way ANOVA followed by Dunnett’s multiple comparison test was used to compare the mean values among the multiple samples. Nonparametric data were analyzed by the Kruskal–Wallis test followed by Dunn’s *post hoc* test. Data were presented as the means ± standard errors (SEMs), with *p* < 0.05 being considered statistically significant.

## 3 Results

### 3.1 Narrowing of the aortic arch causes pathologic hypertrophy in the mouse heart

**FIGURE 1:**
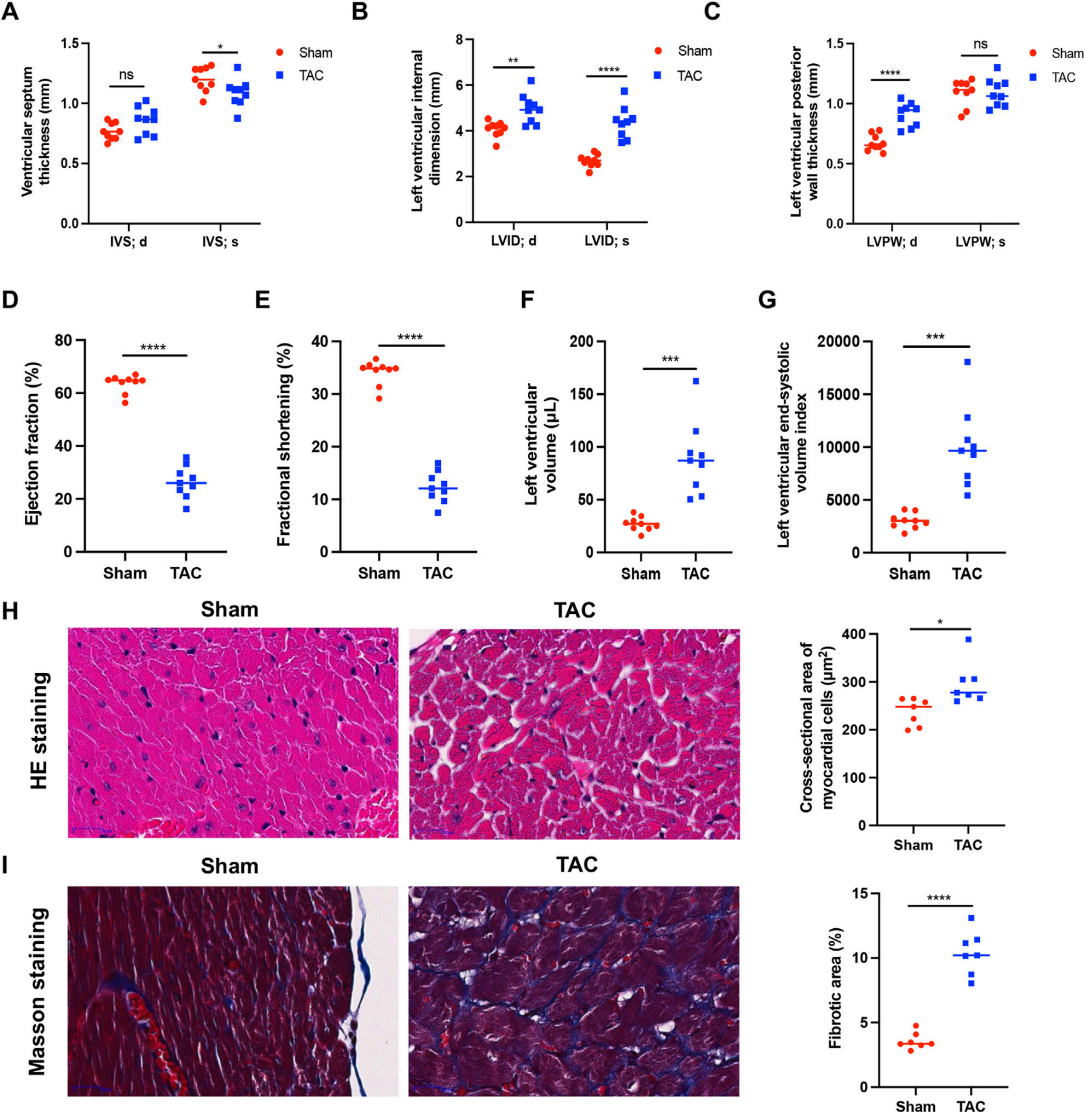
Narrowing of the aortic arch causes pathologic hypertrophy in the mouse heart. **(A)** Left ventricular septal end-diastolic/end-systolic thickness (IVS; d/s). **(B)** Left ventricular end-diastolic/end-systolic internal diameter (LVID; d/s). **(C)** Left ventricular end-diastolic/end-systolic posterior wall thickness (LVPW; d/s). **(D)** Ejection fraction (EF%). **(E)** Fractional shortening (FS%). **(F)** Left ventricular volume. **(G)** Left ventricular end-systolic volume index. **(H)** Representative images of myocardial cells after HE staining (left) and measurement of cross-sectional area (right). **(I)** Representative images of myocardial cells after Masson staining (left) and measurement of fibrotic area (right). The collagen fibers were blue and myofibers were red after staining. Data were represented as mean ± SEM and analyzed by Unpaired *t*-test, *n* = 7–9. ns *p* > 0.05, *p < 0.05, **p < 0.01, ***p < 0.001, ****p < 0.0001 compared with the Sham group.

Mice with normal heart function were randomly divided into two groups: TAC model and sham. We evaluated their cardiac function by B-mode and M-mode ultrasound in the 9th week after surgery. The end-diastolic interventricular septal thickness of the murine hearts did not differ significantly between the two groups; however, in the TAC group, the end-systolic interventricular septal thickness was lower (Figure 1A) and the end-diastolic and end-systolic left ventricular internal diameters were both elevated (Figure 1B). The left ventricular end-diastolic posterior wall thickness was also significantly (*p* < 0.0001) higher in the TAC group than in the sham group, and there was no significant change in end-systolic posterior wall thickness (Figure 1C). Thus, the cardiac structure of the mice in our TAC model significantly expanded.

Ejection fraction and short-axis shortening rate reflect left ventricular emptying and myocardial shortening functions, respectively, and are often used to evaluate the contractile function of the heart. Compared with the sham group, mice in the TAC group had significantly (*p* < 0.0001) reduced ejection fraction (Figure 1D) and short-axis shortening rate (Figure 1E) and increased left ventricular volume (Figure 1F) and left ventricular end-systolic volume index (Figure 1G). These results indicate that the left ventricular systolic function was weakened in the TAC group.

In the 9th week after surgery, murine hearts were prepared for histopathological analyses. The cross-sectional area of cardiomyocytes was observed by H&E staining, which showed that the cardiomyocytes of mice in the TAC group were enlarged compared with those in the sham group (Figure 1H). The collagen concentration increased during myocardial fibrosis, and MASSON staining revealed a significant (*p* < 0.0001) increase in fibrosis in hearts of the TAC group (Figure 1I).

### 3.2 LacCer content was significantly elevated in the hearts of mice in the TAC group

**FIGURE 2:**
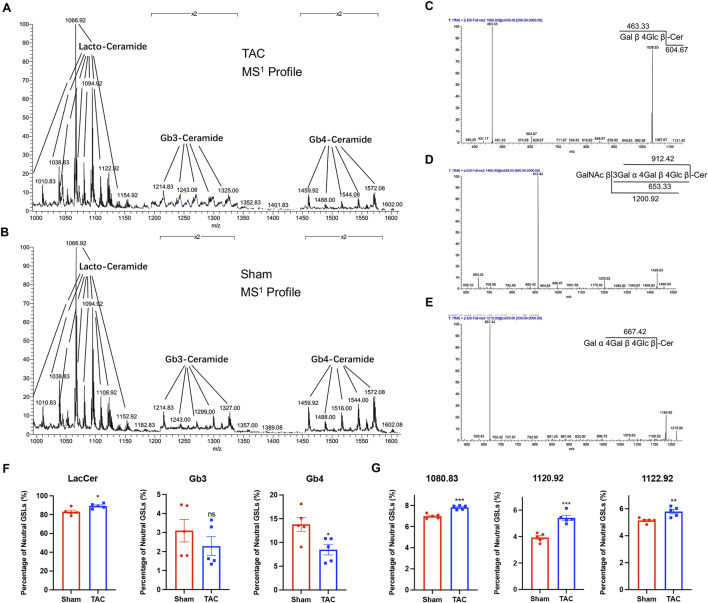
LacCer content was significantly elevated in the hearts of mice in the TAC group. **(A)** MS^1^ profiles of neutral GSLs in the hearts of TAC group mice. **(B)** MS^1^ profiles of neutral GSLs in the hearts of Sham group mice. **(C–E)** MS^2^ profile and ion fragment diagrams of representative ions: **(C)** *m/z* 1066.8, **(D)** *m/z* 1214.9, **(E)** *m/z* 1460.0. **(F)** Relative quantitative analysis of major neutral GSLs in the hearts of TAC and Sham group mice. **(G)** Relative quantitative analysis of *m/z* 1080.83, m*/z* 1120.92, and *m/z* 1122.92 ions in TAC and Sham groups. Data were represented as mean ± SEM and analyzed by Unpaired *t*-test, *n* = 5. ns *p* > 0.05, *p < 0.05, **p < 0.01, ***p < 0.001 compared with the Sham group.

We extracted neutral GSLs from the hearts of mice in the TAC and sham groups and analyzed them by mass spectrometry, obtaining the MS^1^ mass spectra shown in Figures 2A,B (*m/z* = 1000–1600), with the main ions of *m/z* 1066.8, m*/z* 1094.8, m*/z* 1214.9, and *m/z* 1460.0 (all of which are singly charged Na^+^ adduct peaks). The *m/z* 1066.8, *m/z* 1214.9, and *m/z* 1460.0 ions in the MS^1^ profile were subjected to secondary mass spectrometry, and there were two fragments of *m/z* 463.33 and *m/z* 604.67 in the MS^2^ profile of *m/z* 1066.8 (Figure 2C); these were the glycan portion and the ceramide portion of the precursor ion, respectively. The characteristic ion fragment *m/z* 667.42 in the MS^2^ profile of *m/z* 1214.9 was a trisaccharide structure (Figure 2D), and *m/z* 912.42 in the MS^2^ profile of *m/z* 1460.0 was a characteristic ion of tetrasaccharide structure (Figure 2E). Comparison with the GSLs spectral library in our laboratory revealed that *m/z* 1066.8 is C20:0 (the fatty acid in ceramide moiety)-LacCer (GalGlc-Cer), *m/z* 1214.9 is C16:0-Gb3 (GalGalGlc-Cer), and *m/z* 1460.9 is C16:0-Gb4 (GalNAcGalGalGlc-Cer). At last, we counted that the neutral GSLs extracted from our cardiac samples contained mainly LacCer, Gb3, and Gb4.

All the GSLs identified in the different samples were normalized based on peak heights, and the total content of GSLs with different glycan moieties was calculated (Table 2; Figure 2F). The proportion of LacCer in total neutral GSLs was the highest, all above 80%, and the content of LacCer was close to 90% in the TAC group; Gb4 was lower in the TAC group than in the sham group; and the content of Gb3 was the least, less than 5%. Three GSLs, *m/z* 1080.83, *m/z* 1120.92, and *m/z* 1122.92, were all LacCer and higher in the TAC group than in the sham group (Figure 2G). These results indicate that the LacCer content in the hearts was significantly elevated when the mice developed pathological cardiac hypertrophy.

**TABLE 2:** Major types, distribution and content of glycosphingolipids in the heart of mice in TAC and Sham groups.

GSL	Molecular ions	Fatty Acids	Sphingosine	Total GSL (%)	
				TAC	Sham
LacCer	1010.83	16:0	d18:1	89.22	83.13
	1038.83	18:0	d18:1		
	1052.83	19:0	d18:1		
	1066.83	20:0	d18:1		
	1080.83	21:0	d18:1		
	1094.92	22:0	d18:1		
	1108.92	23:0	d18:1		
	1120.92	24:0	d18:2		
	1122.92	24:0	d18:1		
	1152.92	h24:0	d18:1		
Gb3	1214.83	16:0	d18:1	2.29	3.10
	1298.83	22:0	d18:1		
	1325.00	24:1	d18:1		
	1327.00	24:0	d18:1		
Gb4	1460.00	16:0	d18:1	8.5	13.83
	1487.92	18:0	d18:1		
	1516.00	20:0	d18:1		
	1544.00	22:0	d18:1		
	1558.00	23:0	d18:1		
	1570.00	24:1	d18:1		
	1572.00	24:0	d18:1		
	1602.00	h24:0	d18:1		

### 3.3 LacCer content increased significantly after hypertrophy of H9c2 cells

**FIGURE 3:**
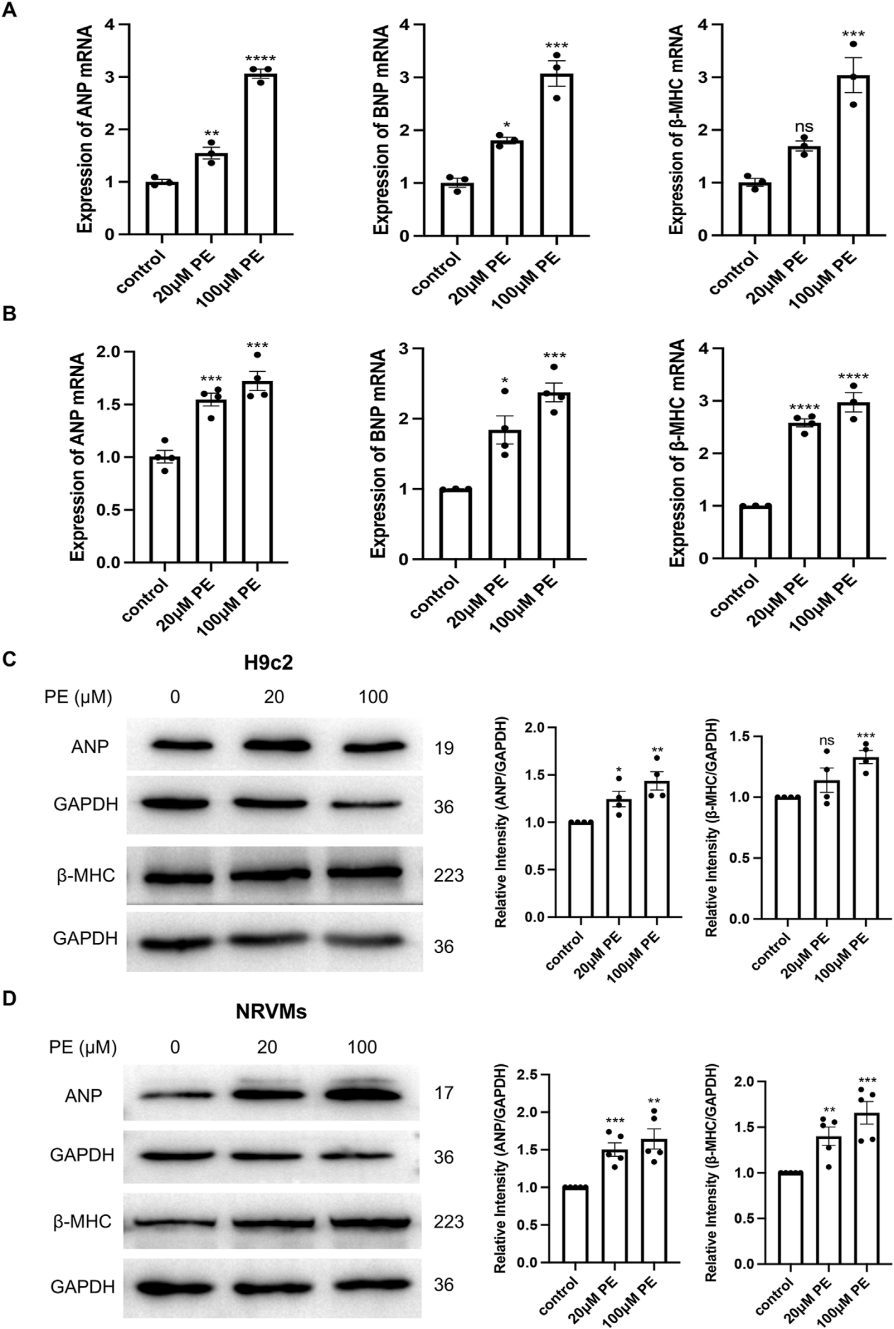
Phenylephrine (PE) stimulation for 48 h causes hypertrophy of H9c2 cells and NRVMs. **(A)** Real-time qPCR analysis of the mRNA expression of *ANP*, *BNP*, and *β-MHC* in H9c2 cells. **(B)** Real-time qPCR analysis of the mRNA expression of *ANP*, *BNP*, and *β-MHC* in NRVMs. Data were represented as mean ± SEM and analyzed by one-way ANOVA followed by Dunnett’s test as a *post hoc* test, *n* ≥ 3. **(C)** Western blot and quantitative analysis of ANP and β-MHC in H9c2 cells. **(D)** Western blot and quantitative analysis of ANP and β-MHC in NRVMs. Protein levels were quantified using grey value analyses by ImageJ software. Data were represented as mean ± SEM and analyzed by Unpaired *t*-test, *n* ≥ 4. ns *p* > 0.05, *p < 0.05, **p < 0.01, ***p < 0.001, ****p < 0.0001 compared with control group.

We stimulated H9c2 cells and NRVMs with 20 μM and 100 μM phenylephrine for 48 h to establish a cardiomyocyte hypertrophy model and subsequently examined the mRNA and protein expression of the hypertrophy-related genes *ANP*, *BNP*, and *β-MHC*. The mRNA expression of *ANP*, *BNP*, and *β-MHC* were significantly (*p* < 0.05) increased after phenylephrine stimulation of H9c2 cells (Figure 3A) and NRVMs (Figure 3B) for 48 h, in a dose-dependent manner. The protein expression of ANP and β-MHC in H9c2 cells and NRVMs were higher in the 100 μM phenylephrine-treated group than in the control group, but the changes of β-MHC in H9c2 cells were not obvious in the 20 μM phenylephrine-treated group (Figures 3C,D). Thus, stimulating cardiomyocytes with 100 μM phenylephrine for 48 h was the most effective condition for inducing hypertrophy.

**FIGURE 4:**
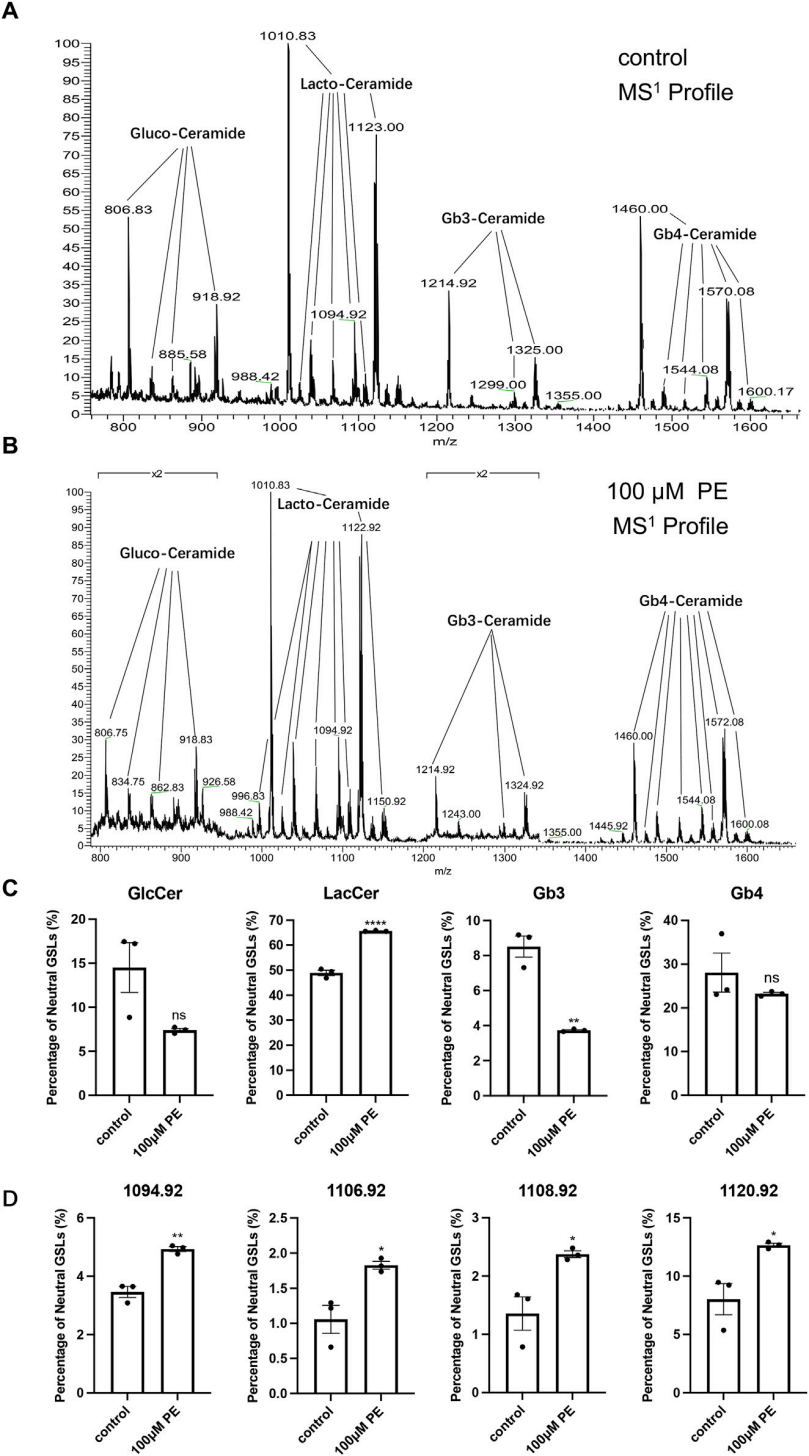
LacCer content in H9c2 cells after 100 µM phenylephrine treatment for 48 h was significantly increased. **(A)** MS^1^ profiles of neutral GSLs in control group. **(B)** MS^1^ profiles of neutral GSLs in phenylephrine-treated group. **(C)** Relative quantitative analysis of major neutral GSLs in H9c2 cells. **(D)** Relative quantitative analysis of *m/z* 1094.92, *m/z* 1106.92, *m/z* 1108.92, and *m/z* 1120.92 ions in control and phenylephrine-treated groups. Data were represented as mean ± SEM and analyzed by Unpaired *t*-test, *n* = 3. ns *p* > 0.05, *p < 0.05, **p < 0.01, ****p < 0.0001 compared with control group.

Considering animal ethics, we used H9c2 cells to analyze the changes in GSLs after cell-stimulated hypertrophy. The MS^1^ profile of neutral GSLs in H9c2 cells is shown in Figures 4A,B, with the main ions of *m/z* = 806.83, *m/z* = 1010.83, *m/z* = 1214.92, and *m/z* = 1460.00. Our qualitative analysis revealed that the main neutral GSLs extracted from the cells were glucosylceramide (GlcCer), LacCer, Gb3, and Gb4. The percentage of each GSL in the total neutral GSLs in H9c2 cells was statistically analyzed (Table 3; Figure 4C). Of the total neutral GSLs, LacCer was the most abundant and was significantly (*p* < 0.0001) higher in the phenylephrine-treated group than in the control group. Next was Gb4, with about 20%–30% of total GSLs; this was not significantly different between the control and phenylephrine-treated groups. Compared with the control group, GlcCer in the phenylephrine-treated group was also not significantly different, but Gb3 was lower. There were four LacCer type GSLs with fatty acyl of different lengths in the phenylephrine-treated group that were higher than in the control group (Figure 4D): *m/z* 1094.92, *m/z* 1106.92, *m/z* 1108.92, and *m/z* 1120.92. These results indicate that hypertrophied H9c2 cells had significantly more LacCer than did normal cells.

**TABLE 3:** Major types, distribution and content of glycosphingolipids in H9c2 cells of Control and Phenylephrine-treated groups.

GSL	Molecular ions	Fatty Acids	Sphingosine	Total GSL (%)	
				Control	100 μM PE
GlcCer	806.75	16:0	d18:1	14.51	7.40
	834.75	18:0	d18:1		
	836.83	h16:0	d18:1		
	862.83	20:0	d18:1		
	916.92	24:1	d18:1		
	918.83	24:0	d18:1		
LacCer	996.83	15:0	d18:1	48.91	65.66
	1010.83	16:0	d18:1		
	1024.83	17:0	d18:1		
	1038.83	18:0	d18:1		
	1066.83	20:0	d18:1		
	1094.92	22:0	d18:1		
	1106.92	23:0	d18:2		
	1108.92	23:0	d18:1		
	1120.92	24:0	d18:2		
	1122.92	24:0	d18:1		
	1136.92	25:0	d18:1		
	1150.92	h24:0	d18:1		
Gb3	1214.92	16:0	d18:1	8.55	3.73
	1325.00	24:1	d18:1		
	1327.00	24:0	d18:1		
Gb4	1460.00	16:0	d18:1	28.24	23.21
	1474.00	17:0	d18:1		
	1476.00	h15:0	d18:1		
	1488.00	18:0	d18:1		
	1490.00	h16:0	d18:1		
	1516.08	20:0	d18:1		
	1544.08	22:0	d18:1		
	1558.08	23:0	d18:1		
	1570.08	24:1	d18:1		
	1572.08	24:0	d18:1		
	1586.08	25:0	d18:1		
	1600.08	h24:0	d18:1		

### 3.4 Inhibition of GSLs synthesis suppresses expression of hypertrophy-related genes in NRVMs

**FIGURE 5:**
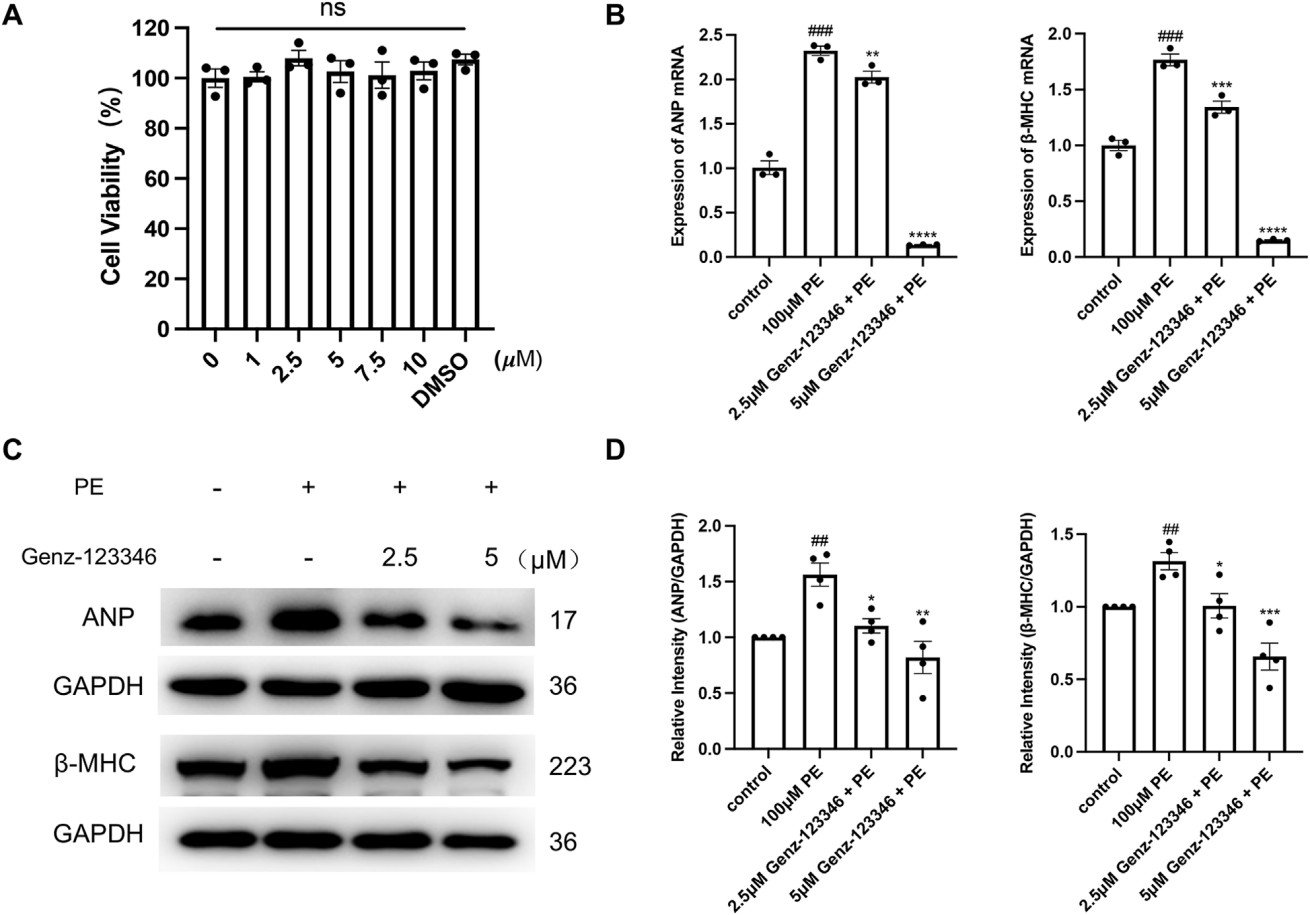
Inhibition of GSLs synthesis reduces the expression of hypertrophy-related genes in NRVMs. Cells were pretreated with 2.5 μM or 5 μM Genz-123346 for 60 h and then treated with 100 μM phenylephrine for 48 h **(A)** NRVMs viability was detected after 60 h treatment with varying Genz-123346 doses. Data were represented as mean ± SEM and analyzed by one-way ANOVA followed by Dunnett’s test as a *post hoc* test, *n* = 3. ns *p* > 0.05 compared with control group. **(B)** Real-time qPCR analysis of the mRNA expression of *ANP* and *β-MHC* (*n* = 3). **(C, D)** Western blot and quantitative analysis of ANP and β-MHC (*n* = 4). Protein levels were quantified using grey value analyses by ImageJ software. Data were represented as mean ± SEM and analyzed by Unpaired *t*-test; ##*p* < 0.01, ###*p* < 0.001 compared with control group. Data were represented as mean ± SEM and analyzed by one-way ANOVA followed by Dunnett’s test as a *post hoc* test; *p < 0.05, **p < 0.01, ***p < 0.001, ****p < 0.0001 compared with phenylephrine-treated group.

Genz-123346 is a specific GlcCer synthase inhibitor that blocks the conversion of ceramide to GlcCer and thus inhibits LacCer production. First, we examined the effect of Genz-123346 on the viability of H9c2 cells (Supplementary Figure 1A) and NRVMs (Figure 5A) by CCK-8 assays, and their viability was not affected by Genz-123346 concentrations below 5 μM for 60 h. Moreover, treatment of H9c2 cells with 5 μM Genz-123346 for 60 h inhibited the production of GSLs in the cells (Supplementary Figures 1B, C). To investigate whether inhibition of GSL synthesis reverses cardiomyocyte hypertrophy, NRVMs were divided into the following groups: control, phenylephrine, and Genz-123346 pretreatment (2.5 or 5 μM Genz-123346 for 60 h). After 48 h of culturing with phenylephrine, cells were used for real-time qPCR and Western blotting. Real-time qPCR results showed that the mRNA expression of *ANP* and *β-MHC* in NRVMs were significantly (*p* < 0.001) increased after 48 h of induction with 100 μM phenylephrine and decreased in cells pretreated with Genz-123346 for 60 h (Figure 5B); furthermore, the inhibitor exhibited increased effect at 5 μM concentration than 2.5 μM Western blot results were consistent with the gene transcription results; the protein expression of ANP and β-MHC were higher in cells without the addition of Genz-123346 than in the control group, whereas the expression of both was decreased in cells pretreated with Genz-123346 (Figures 5C,D). Again, the effect was better in the group with 5 μM of the inhibitor. Thus, under our experimental conditions, inhibition of GSL synthesis reduces the expression of hypertrophy-related genes in NRVMs in an inhibitor dose-dependent manner.

### 3.5 Inhibition of GSLs synthesis attenuates fibrosis and reduces apoptosis in NRVMs

**FIGURE 6:**
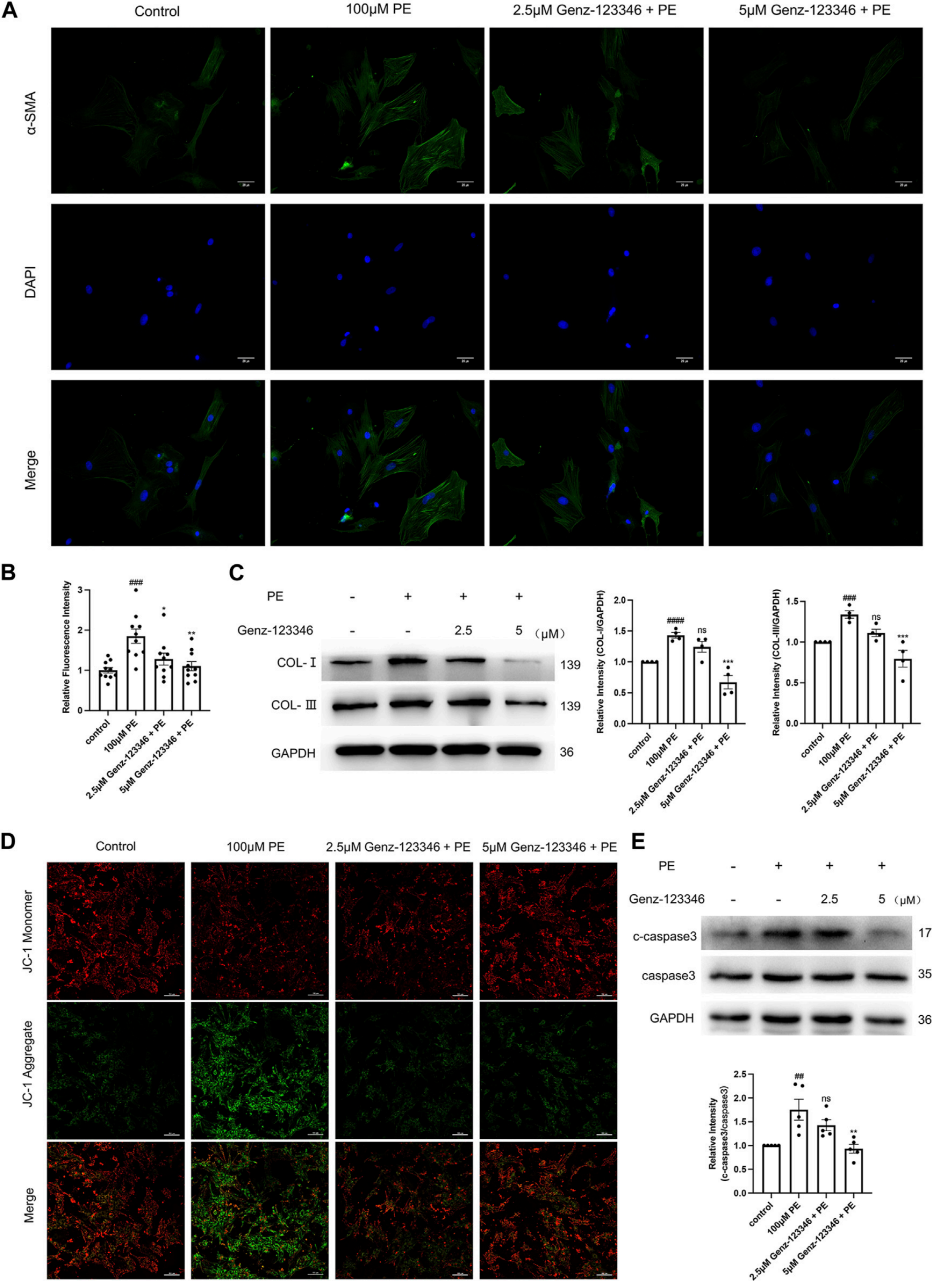
Inhibition of GSLs synthesis attenuates fibrosis and reduces apoptosis in NRVMs. Cells were pretreated with 2.5 μM or 5 μM Genz-123346 for 60 h and then treated with 100 μM phenylephrine for 48 h. **(A)** Representative images of the cells in each group after immunofluorescence staining with α-SMA (images were taken at ×200 magnification). Bar = 20 μm. **(B)** Relative quantitative analysis of α-SMA mean fluorescence intensity (*n* = 10). **(C)** Western blot and quantitative analysis of type I and type III collagen (*n* = 4). **(D)** Representative images of the cells in each group after JC-1 staining (images were taken at ×200 magnification). Bar = 20 μm. **(E)** Western blot and quantitative analysis of cleaved-caspase3 (c-caspase3) and caspase3 (*n* = 5). Protein levels were quantified using grey value analyses by ImageJ software. Data were represented as mean ± SEM and analyzed by Unpaired *t*-test; ##*p* < 0.01, ###*p* < 0.001, ####*p* < 0.0001 compared with control group. Data were represented as mean ± SEM and analyzed by one-way ANOVA followed by Dunnett’s test as a *post hoc* test; ns *p* > 0.05, *p < 0.05, **p < 0.01, ***p < 0.001 compared with phenylephrine-treated group.

α-SMA is a marker of fibrosis. We performed immunofluorescence staining of cardiomyocytes with an α-SMA antibody (Figure 6A) and relative quantification of the average fluorescence intensity of the cells (Figure 6B). The fluorescence intensity after phenylephrine stimulation was higher than that of the control group, whereas in the Genz-123346 pretreated group, the fluorescence intensity after stimulation decreased. Also, the extent of the decrease was more pronounced at the higher concentration of Genz-123346 treatment. Next, we detected the expression of type I and type III collagen in the cells of each group by Western blotting (Figure 6C). The expression of type I and type III collagen increased after phenylephrine treatment compared with the control group, and the expression of both collagen types gradually decreased with the increase of Genz-123346 concentration compared with the phenylephrine group.

Decreased mitochondrial membrane potential is a hallmark of early apoptosis, and to detect apoptosis, the mitochondrial membrane potential was assessed by JC-1 staining (Figure 6D). The red fluorescence of the cells was weakened and the green fluorescence was enhanced after phenylephrine stimulation compared with the control group, suggesting that the mitochondrial membrane potential was reduced. The mitochondrial membrane potential of cells in the Genz-123346-treated group rebounded compared with phenylephrine-induced cells. Next, protein expression of the apoptosis execution protein caspase3 was examined by Western blotting, and the ratio of cleaved-caspase3/caspase3 was elevated in phenylephrine-induced cells and progressively decreased in cells pretreated with Genz-123346 (Figure 6E). These results suggest that inhibition of GSL synthesis attenuated fibrosis and apoptosis in cardiomyocytes.

### 3.6 Inhibition of GSLs synthesis reduces intracellular ROS levels by increasing Sirt3 protein expression

**FIGURE 7:**
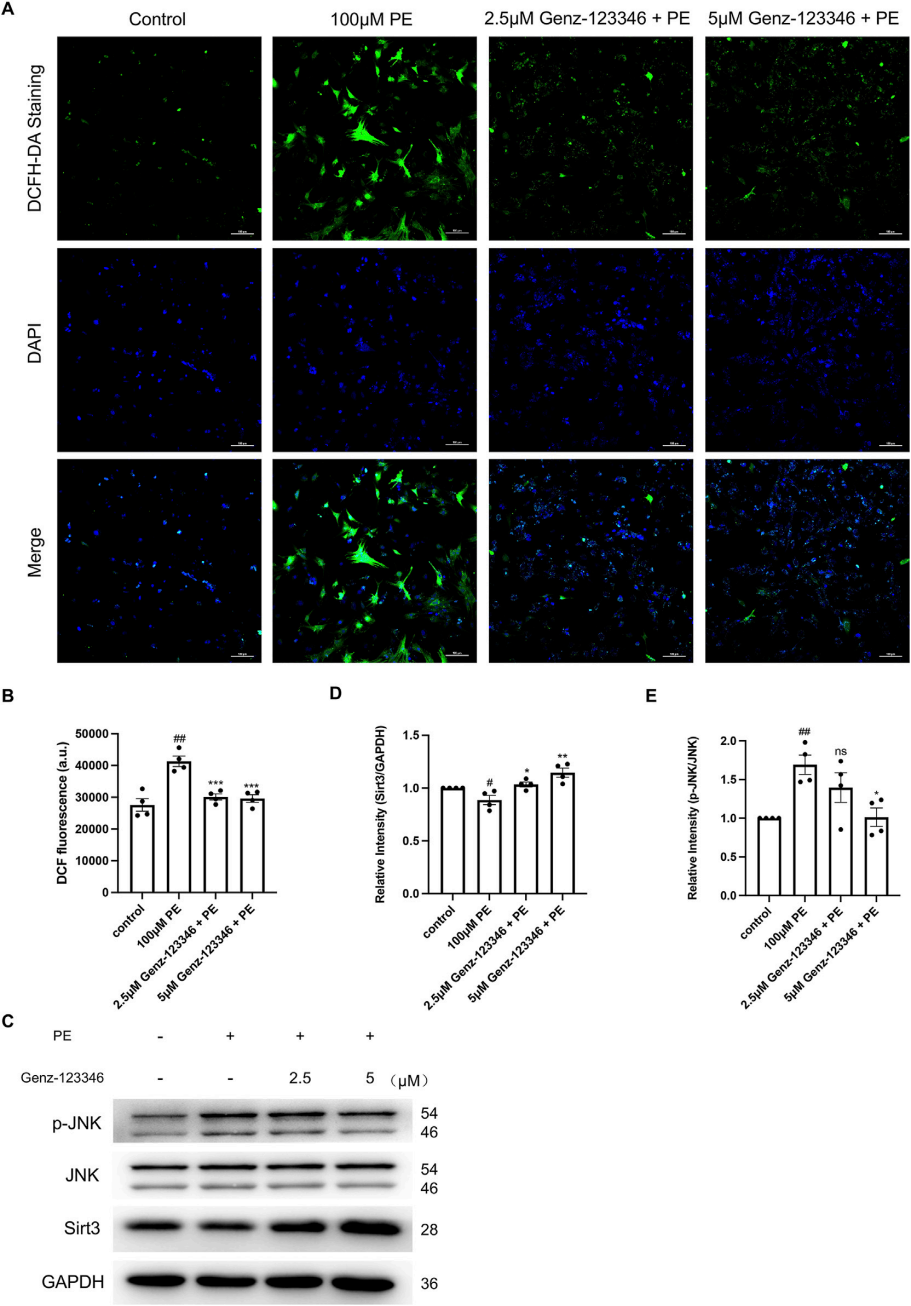
Inhibition of GSLs synthesis reduces intracellular ROS levels by increasing Sirt3 protein expression, which in turn reduces the phosphorylation of JNK. NRVMs were pretreated with 2.5 μM or 5 μM Genz-123346 for 60 h and then treated with 100 μM phenylephrine for 48 h. **(A)** Representative images of the cells in each group after DCFH-DA staining (images were taken at ×200 magnification). Bar = 20 μm. **(B)** Fluorescence intensity of intracellular DCF detected with multifunctional enzyme marker (*n* = 4). **(C–E)** Western blot and quantitative analysis of Sirt3, JNK, and p-JNK (*n* = 4). Protein levels were quantified using grey value analyses by ImageJ software. Data were represented as mean ± SEM and analyzed by Unpaired *t*-test; #*p* < 0.05, ##*p* < 0.01 compared with control group. Data were represented as mean ± SEM and analyzed by one-way ANOVA followed by Dunnett’s test as a *post hoc* test; ns *p* > 0.05, *p < 0.05, **p < 0.01, ***p < 0.001 compared with phenylephrine-treated group.

We detected intracellular ROS levels using a DCFH-DA fluorescent probe (Figure 7A), and the fluorescence intensity of intracellular DCF was directly detected using an enzyme marker (Figure 7B). The fluorescence intensity of DCF of phenylephrine-induced NRVMs was higher than that of the control group, indicating increased ROS production, which was attenuated by pretreatment with Genz-123346. The mitochondrial deacetylase Sirt3 has been shown to attenuate cardiomyocyte hypertrophy by reducing ROS production, so we examined the intracellular protein expression of Sirt3 by Western blotting. Sirt3 protein expression was suppressed in phenylephrine-stimulated cells and progressively increased in cells pretreated with Genz-123346 (Figures 7C,D).

Oxidative stress due to ROS accumulation can activate the MAPK signaling pathway and thus cause cellular hypertrophy, and JNK as a stress kinase has been demonstrated to be upregulated during stress overload. Western blot analysis indicated an increase in the phosphorylation level of JNK after phenylephrine treatment and another gradual decrease in Genz-123346-pretreated cells (Figures 7C,E). Together, these results suggest that inhibition of GSL synthesis reduces intracellular ROS production by increasing the expression of Sirt3 protein in the mitochondria, which in turn reduces the phosphorylation level of JNK.

### 3.7 Inhibition of GSLs synthesis restores autophagic homeostasis

**FIGURE 8:**
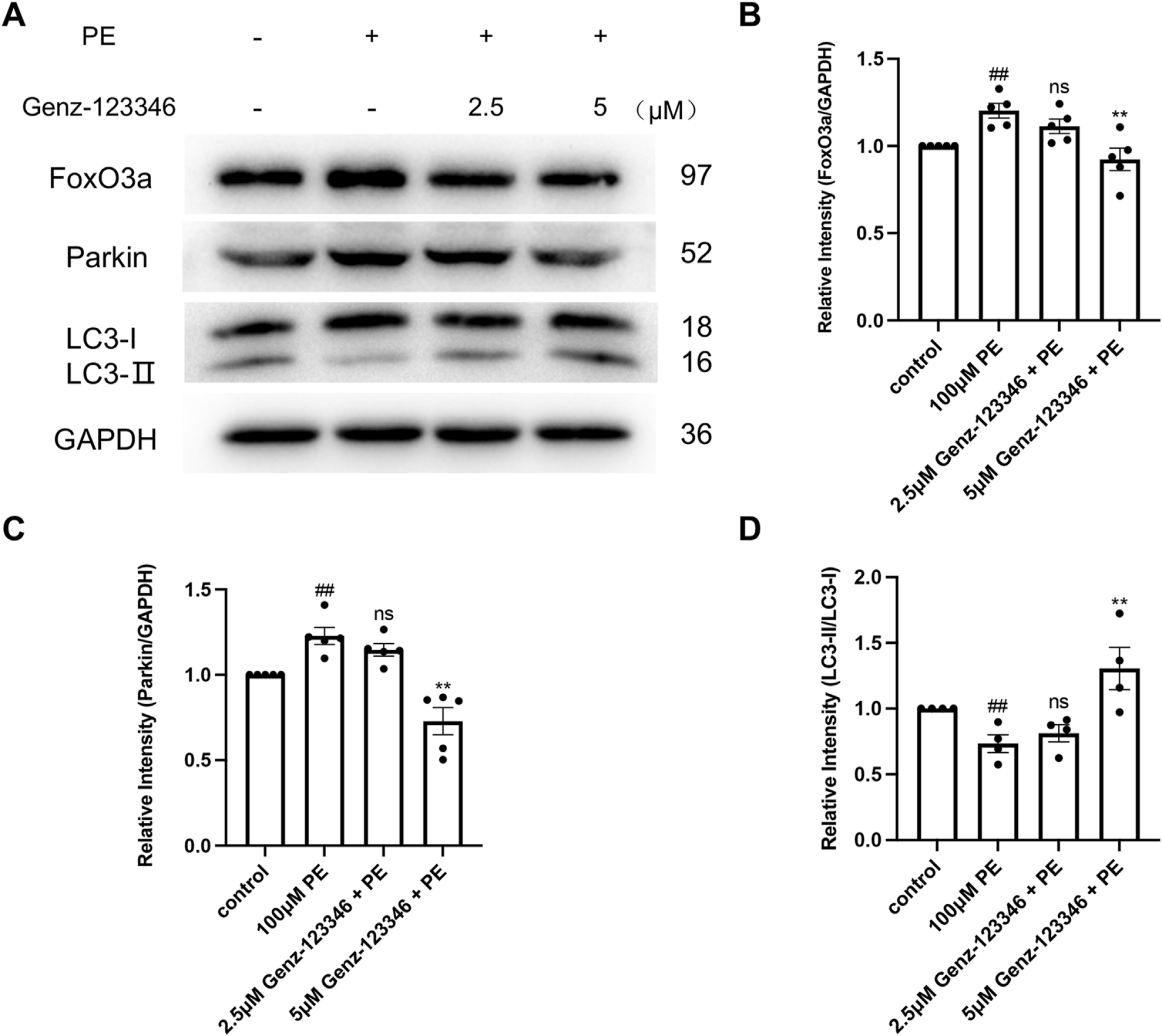
Inhibition of GSLs synthesis restores autophagic homeostasis. NRVMs were pretreated with 2.5 μM or 5 μM Genz-123346 for 60 h and then treated with 100 μM phenylephrine for 48 h **(A–D)** Western blot and quantitative analysis of FoxO3a, Parkin, and LC3. Protein levels were quantified using grey value analyses by ImageJ software. *n* ≥ 4. Data were represented as mean ± SEM and analyzed by Unpaired *t*-test; ##*p* < 0.05 compared with control group. Data were represented as mean ± SEM and analyzed by one-way ANOVA followed by Dunnett’s test as a *post hoc* test; Nonparametric data were analyzed by the Kruskal–Wallis test followed by Dunn’s *post hoc* test; ns *p* > 0.05, **p < 0.01 compared with phenylephrine-treated group.

Autophagy plays an important role in maintaining cellular homeostasis. Western blot analysis showed that the expression of FoxO3a and Parkin in NRVMs after phenylephrine treatment was increased and then decreased in the cells pretreated with 5 μM Genz-123346 (Figures 8A–C). The ratio of LC3-II/I in the cells of the phenylephrine-treated group was lower than that of the control group and then gradually increased after Genz-123346 pretreatment compared with the phenylephrine group (Figures 8A,D). Thus, hypertrophied NRVMs exhibited abnormal autophagy, but the autophagy homeostasis tended to recover after inhibiting GSLs synthesis.

## 4 Discussion

Unlike lipids used for energy and structure, sphingolipids, ceramides, and sphingomyelins respond to specific stimuli and are part of signaling pathways (Hannun and Obeid, 2018). LacCer is a GSLs thought to have a variety of bioactivities and is involved in the pathogenesis of diseases such as colorectal cancer, diabetes mellitus, Parkinson’s disease, and systemic lupus erythematosus. A strong correlation between LacCer levels and arterial stiffness has been reported, with elevated arterial stiffness indicating increased atherosclerosis, which is associated with an increased risk of cardiovascular disease in adults (Jung et al., 2015). A high-fat, high-cholesterol diet was administered to 12-week-old ApoE^−/−^ mice (atherosclerotic mouse model), and serial cardiac ultrasound tests at 20 and 36 weeks revealed a highly positive correlation between LacCer levels and pulse wave velocity (PWV). After treatment with D-PDMP, which inhibits GSL synthesis, the levels of LacCer and PWV decreased in parallel groups of mice (Chatterjee et al., 2014). Another study in the same model found that the treatment of mice with D-PDMP could not only reverse atherosclerosis but also significantly improve cardiac hypertrophy (Mishra et al., 2015). These observations were confirmed in a human study with a 3-year follow-up of overweight and normal-weight middle-aged subjects; the overweight groups had increased L-leucine, LacCer, and brachial-ankle PVW, and changes in LacCer independently predicted changes in brachial-ankle PWV (Kim et al., 2015).

In our experiments, LacCer (the glycan chain moiety contains two monosaccharides) was found to be the most abundant class of neutral GSLs, and its content was significantly increased in the hearts of cardiac hypertrophied mice, whereas Gb3 and Gb4 (the glycan chain moiety contains three and four monosaccharides, respectively) were decreased compared with controls. LacCer is a substrate for the synthesis of Gb3 and Gb4. Higher LacCer may be due to either increased synthesis of or decreased metabolism of LacCer, and with the decrease of Gb3 and Gb4, but how this reduction of Gb3 and Gb4 affects cardiac hypertrophy requires further research. Similar results were seen in the H9c2 cell line (we performed this experiment only on the H9c2 cell line for animal ethical reasons, given the huge amount of cells required for GSL extraction). In the H9c2 cells, the increase in LacCer content was accompanied by the detection of approximately 10% GlcCer in the phenylephrine-stimulated hypertrophied H9c2 cells, which was almost undetectable in mouse cardiac extracts. This is likely attributable to the rapid growth of the cells and the high synthesis of GSLs. This makes us ponder whether the inhibition of LacCer, or in other words, the synthesis of whole GSLs, delays myocardial hypertrophy.

In our NRVMs hypertrophy model stimulated by phenylephrine, we observed a decrease in cell hypertrophy after pretreatment with the GSLs synthesis inhibitor Genz-123346. We used Genz-123346 to pretreat NRVMs for 48 h and then treated them with phenylephrine for 48 h in the pre-experiment. The mRNA of *ANP* and *β-MHC* in the Genz-123346-pretreatment group was reduced and close to those in the control group (Supplementary Figure 2). However, pretreatment with inhibitors for 60 h showed the more significant and stable inhibitory effect on hypertrophy in myocardial cells. So, 60 h was used in subsequent experiments. We also observed that the mRNA of *ANP* and *β-MHC* in the Genz-123346-pretreated group was significantly lower than that in the control group. This may be due to the higher concentration and longer duration of action of Genz-123346, which may affect other metabolic processes in the cells. If the processing time is further prolonged, the long-term lack of GSLs results in issues with cell growth. Pathological cardiac hypertrophy is generally accompanied by fibrosis and apoptosis of cardiomyocytes (Nakamura and Sadoshima, 2018), and these two processes are also alleviated to varying degrees by inhibitor pretreatment. This suggests that Genz-123346 inhibited the synthesis of GSLs in cardiomyocytes, which subsequently inhibited the phenylephrine-stimulated cellular hypertrophy phenomenon while attenuating the accompanying fibrosis and cardiomyocyte apoptosis.

In related projects in our laboratory, sequencing of murine hearts in the TAC and sham groups suggests that genes significantly downregulated in the TAC group compared with the sham group are associated with such biological processes as glycoprotein and glucose metabolism, and endoplasmic reticulum stress response. GSLs are mainly synthesized in the endoplasmic reticulum and Golgi apparatus (Mandon et al., 1992; Hirschberg et al., 1993), but Ardail et al. (2001) found GlcCer and LacCer in the outer mitochondrial membrane, a phenomenon that may be related to mitochondria-associated endoplasmic reticulum membranes. These membranes contain UDP-glucose ceramide glucosyltransferase, LacCer synthetase, and sialyltransferase (Ardail et al., 2003), and ceramide can also be produced in mitochondria (Hernández-Corbacho et al., 2017). Thus, GSLs can be produced in mitochondria (Ardail et al., 2003), and the abnormal accumulation of LacCer in mitochondria is considered to be an important factor leading to mitochondrial dysfunction. This has been confirmed in a model of streptozotocin-induced type I diabetic mice. Addition of exogenous LacCer to the baseline mitochondria can reproduce the phenotype of diabetes (Novgorodov et al., 2016). LacCer directly inhibits isolated mitochondrial respiratory chain complexes I and IV (García-Ruiz et al., 2000), resulting in increased mitochondrial ROS production and elevated levels of oxidative stress in the hearts of streptozotocin-induced diabetic mice (Dabkowski et al., 2009; Baseler et al., 2013); this has been implicated as a potential cause of diabetic cardiomyopathy. Sirt3 is an NAD (+) - dependent deacetylase in mitochondria that participates in all aspects of mitochondrial metabolism, including synthesis and movement. It regulates ATP production and mitochondrial adaptive response to stress and plays an important role in improving mitochondrial dysfunction (Zhang et al., 2020; Morigi et al., 2018). Huan Wang et al. demonstrated that supplementing nicotinamide mononucleoside can significantly increase NAD^+^ level, NAD^+^/NADH ratio, and Sirt3 expression in mesenchymal stem cells (MSCs) (Wang et al., 2022). Overexpression of Sirt3 alleviates mitochondrial dysfunction and restores age-related phenotypic features in late passage MSCs (Wang et al., 2022). Research has shown that exercise training increases the content of Sirt3 protein in hippocampal mitochondria of APP/PS1 transgenic (Tg) mice, reduces the acetylation of OGG1 and MnSOD, and reduces ROS production and mtDNA oxidative damage in Tg mice, thereby preventing Alzheimer’s disease-related mitochondrial dysfunction and phenotype deterioration (Bo et al., 2014). In our experiments, we observed elevated Sirt3 protein expression in mitochondria, decreased ROS content in cells, and reduced phosphorylation levels of JNK kinase after inhibition of GSLs synthesis. Therefore, we speculate that inhibiting the synthesis of GSLs can improve mitochondrial function by increasing the expression of Sirt3 in mitochondria, reducing the production of ROS, and inhibiting JNK phosphorylation, ultimately alleviating myocardial cell hypertrophy.

A balanced level of autophagy is necessary for the maintenance of cellular homeostasis; the absence or overactivation of autophagy can adversely affect the cell, and appropriate levels of autophagy can remove damaged mitochondria and reduce ROS production in the cell. GSLs have also been shown to play a role in aberrant autophagy. For example, in an analysis of lung tissue sections from patients with chronic obstructive pulmonary disease, LacCer levels in the lungs were positively correlated with the severity of emphysema and accompanied by increased p62 expression. Treatment with the GSL synthesis inhibitor D-PDMP decreased both LacCer and p62 expression and reversed cigarette smoke-induced chronic obstructive pulmonary disease emphysema (Bodas et al., 2015). In our laboratory’s previous research, we found that overexpression of C18 ceramide in glioma cells triggers cellular autophagy (Wang et al., 2017). The mitochondrial autophagy pathway can be divided into ubiquitin-dependent and non-ubiquitin-dependent pathways. The PINK1/Parkin pathway is a ubiquitin-dependent pathway, and the regulatory mechanism mediated by this pathway is the classical mode of mitochondrial autophagy (Ding et al., 2021). When the mitochondrial membrane potential decreases, the precursor protein of PINK1, retained on the outer membrane of mitochondria, undergoes autophosphorylation and becomes activated. Activated PINK1 can then recruit Parkin from the cytoplasm to the outer membrane of mitochondria and activate its E3 ubiquitin enzyme activity. Parkin ubiquitinates outer mitochondria membrane protein, and the ubiquitin chain is phosphorylated by PINK1. These phosphorylated ubiquitin-modified outer membrane proteins are recognized by autophagy adaptor proteins (such as p62, NDP52, and OPTN), thereby initiating autophagy. Yang et al. (2024) found that in rats with painful diabetic neuropathy, mitochondria were damaged, mitochondrial autophagy was inhibited, and Sirt3 expression was inhibited. Overexpression of Sirt3 restored inhibited autophagy by activating the FoxO3a-PINK1-Parkin signaling pathway, improved damaged mitochondria, and reversed abnormal pain and nociceptor overexcitation. In our study, we found that the expression of FoxO3a and Parkin was higher in phenylephrine-stimulated cells than that of the control group, suggesting that phenylephrine stimulation may induce mitochondrial autophagy through the FoxO3a-PINK1 Parkin signaling pathway. However, changes of LC3 protein suggest that downstream steps of mitochondrial autophagy may be inhibited after phenylephrine stimulation. We think that this is due to the inability of autophagy adaptor proteins to recognize phosphorylated ubiquitin-modified outer membrane proteins, which prevent damaged mitochondria from being effectively cleared. Inhibition of GSLs synthesis restores the integrity of the mitochondrial autophagy process. This intriguing research point may give us new clues to study drugs related to alleviating cardiac hypertrophy. Furthermore, the opposing changes in Sirt3 and FoxO3a suggest that Sirt3 may not be a key upstream factor of FoxO3a in this pathway.

Overall, the present study found the changes in the structural composition of GSLs, manifested as elevated LacCer, were observed in cardiac tissues of mice with cardiac hypertrophy and in cardiac hypertrophic cell lines. Furthermore, inhibition of GSLs synthesis alleviated primary cardiomyocyte hypertrophy. The mechanism for this may be that inhibition of GSLs synthesis reduces intracellular ROS and inhibits the downstream JNK signaling pathway by increasing the expression of Sirt3 in the mitochondria, as well as restoring mitochondrial autophagy homeostasis, ultimately alleviating the symptoms of cardiomyocyte hypertrophy.

## Data Availability

The raw data supporting the conclusions of this article will be made available by the authors, without undue reservation.

## Abbreviations

GSL, glycosphingolipid; LacCer, lactosylceramide; NRVMs, neonatal rat left ventricular myocytes; ROS, reactive oxygen species; PE, phenylephrine; SD, Sprague-Dawley; TAC, transverse aortic constriction; FBS, fetal bovine serum; HE, hematoxylin and eosin; mRNA, messenger ribonucleic acid; PAGE, polyacrylamide gel electrophoresis; PBS, phosphate buffer saline; GlcCer, glucosylceramide; Gb3, globotriaosylceramide; Gb4, globotetraosylceramide; ANP, atrial natriuretic peptide; BNP, brain natriuretic peptide; β-MHC, Myosin heavy chain; MAPK, mitogen-activated protein kinase; α-SMA, α-smooth muscle actin; JC-1, 5,5′,6,6′-Tetrachloro-1,1′,3,3′-tetraethyl-benzimidazolylcarbocyanine iodide; DCFH-DA, 2′,7′-Dichlorodihydrofluorescein diacetate.
