# Supplementary material for: Inhibiting glycosphingolipids alleviates cardiac hypertrophy by reducing reactive oxygen species and restoring autophagic homeostasis
Source: Front Pharmacol. 2024 Oct 1;15:1409625. doi: 10.3389/fphar.2024.1409625 (PMC11474036; doi:10.3389/fphar.2024.1409625)
Supplement: Supplementary file 1 [file DataSheet1.pdf]

Supplementary Materials

Genz-123346 inhibits the production of GSLs in H9c2 cells.

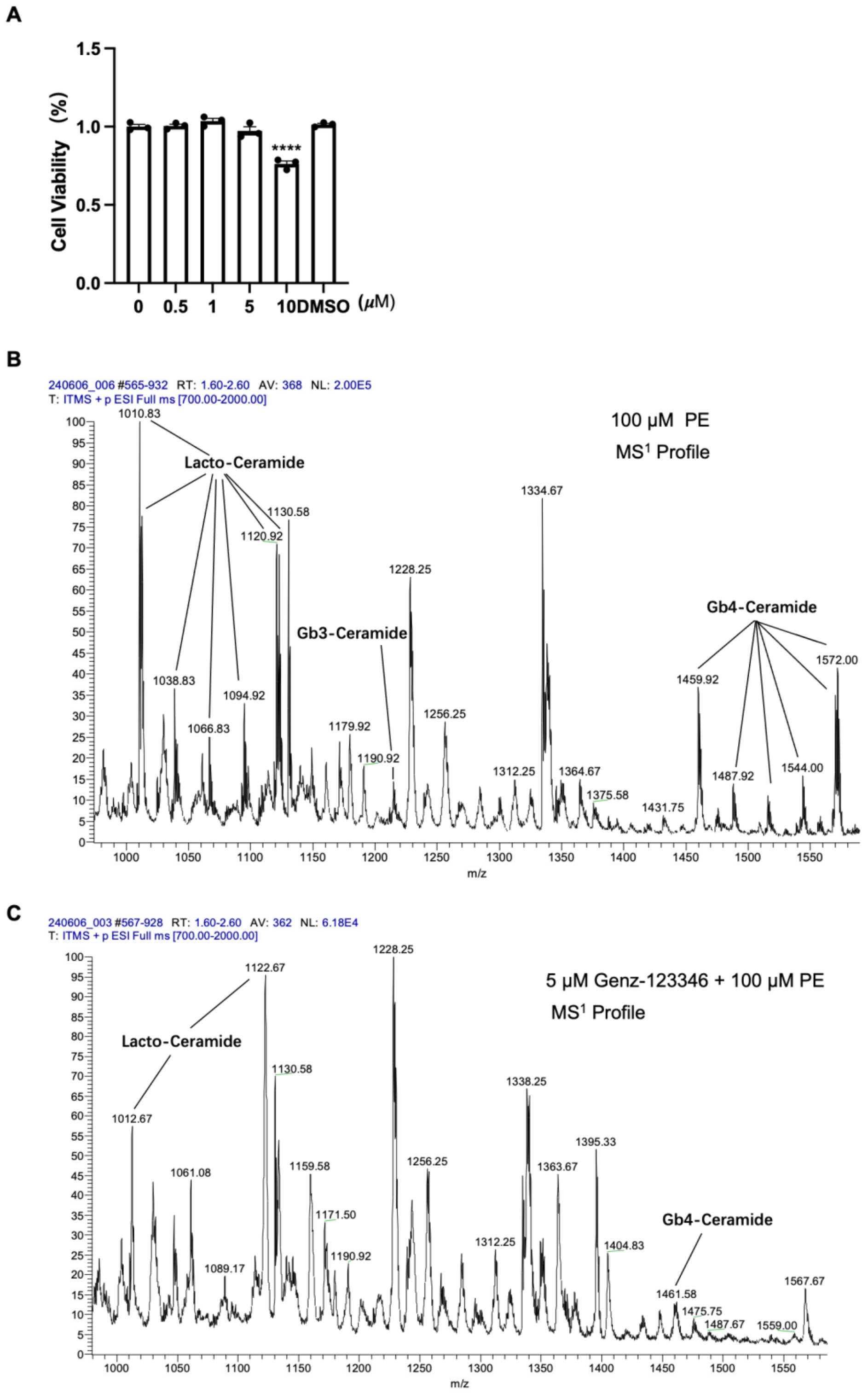

Supplementary Figure 1. Genz-123346 inhibits the production of GSLs in H9c2 cells. Cells were pretreated with 5 μM Genz-123346 for 60 h and then treated with 100 μM phenylephrine for 48 h. (A) H9c2 cells viability was detected after 60 h treatment with varying Genz-123346 doses. $n = 3$. Data were represented as mean ± SEM and analyzed by one-way ANOVA followed by Dunnett's test as a post hoc test, **** $P < 0.0001$ compared with control group. (**B**) MS^1 profiles of neutral GSLs in phenylephrine-treated group. (**C**) MS^1 profiles of neutral GSLs in Genz-123346-pretreated group.

Inhibition of GSLs synthesis reduces the expression of hypertrophy-related genes in NRVMs.

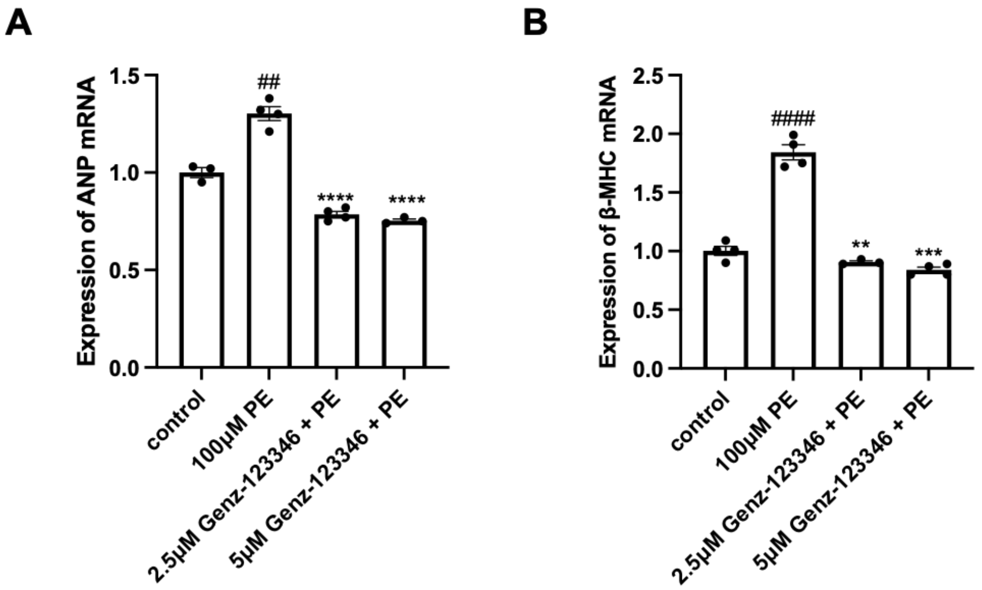

Supplementary Figure 2. Inhibition of GSLs synthesis reduces the expression of hypertrophy-related genes in NRVMs. Cells were pretreated with 2.5 μM or 5 μM Genz-123346 for 48 h and then treated with 100 μM phenylephrine for 48 h. (A) Real-time qPCR analysis of the mRNA expression of *ANP*. (B) Real-time qPCR analysis of the mRNA expression of *β-MHC*. $n \geq 3$. Data were represented as mean ± SEM and analyzed by Unpaired t-test; ##$P < 0.01$, ####$P < 0.0001$ compared with control group; Data were represented as mean ± SEM and analyzed by one-way ANOVA followed by Dunnett's test as a post hoc test; **$P < 0.01$, ***$P < 0.001$, ****$P < 0.0001$ compared with phenylephrine-treated group.
